# Reveal the kernel dehydration mechanisms in maize based on proteomic and metabolomic analysis

**DOI:** 10.1186/s12870-023-04692-z

**Published:** 2024-01-02

**Authors:** Hao Zhang, Xiaonan Gou, Liangchuan Ma, Xiaojun Zhang, Jianzhou Qu, Xiaoyue Wang, Wenjie Huang, Shijuan Yan, Xinghua Zhang, Jiquan Xue, Shutu Xu

**Affiliations:** 1 Key Laboratory of Biology and Genetic Improvement of Maize in Arid Area of Northwest Region, Ministry of Agriculture and Rural Affairs, College of Agronomy, Northwest A&F University, Shaanxi, 712100 Yangling China; 2 Maize Engineering Technology Research Centre, Shaanxi, 712100 Yangling China; 3 Guangdong Key Laboratory for Crop Germplasm Resources Preservation and Utilization, Agro-Biological Gene Research Center, Guangdong Academy of Agricultural Sciences, Guangdong, 510640 Guangzhou China

**Keywords:** Kernel dehydration rate (KDR), Kernel moisture content (KMC), Proteomics, Metabolomics, Maize

## Abstract

**Background:**

Kernel dehydration is an important factor for the mechanized harvest in maize. Kernel moisture content (KMC) and kernel dehydration rate (KDR) are important indicators for kernel dehydration. Although quantitative trait loci and genes related to KMC have been identified, where most of them only focus on the KMC at harvest, these are still far from sufficient to explain all genetic variations, and the relevant regulatory mechanisms are still unclear. In this study, we tried to reveal the key proteins and metabolites related to kernel dehydration in proteome and metabolome levels. Moreover, we preliminarily explored the relevant metabolic pathways that affect kernel dehydration combined proteome and metabolome. These results could accelerate the development of further mechanized maize technologies.

**Results:**

In this study, three maize inbred lines (KB182, KB207, and KB020) with different KMC and KDR were subjected to proteomic analysis 35, 42, and 49 days after pollination (DAP). In total, 8,358 proteins were quantified, and 2,779 of them were differentially expressed proteins in different inbred lines or at different stages. By comparative analysis, K-means cluster, and weighted gene co-expression network analysis based on the proteome data, some important proteins were identified, which are involved in carbohydrate metabolism, stress and defense response, lipid metabolism, and seed development. Through metabolomics analysis of KB182 and KB020 kernels at 42 DAP, 18 significantly different metabolites, including glucose, fructose, proline, and glycerol, were identified.

**Conclusions:**

In sum, we inferred that kernel dehydration could be regulated through carbohydrate metabolism, antioxidant systems, and late embryogenesis abundant protein and heat shock protein expression, all of which were considered as important regulatory factors during kernel dehydration process. These results shed light on kernel dehydration and provide new insights into developing cultivars with low moisture content.

**Supplementary Information:**

The online version contains supplementary material available at 10.1186/s12870-023-04692-z.

## Background

Maize (*Zea mays L*.) is one of the main crops cultivated globally, and whole-process mechanization is becoming more common in maize production. However, most commercial varieties in China are not well suited for mechanical harvest with the kernel, which is the major bottleneck for maize production [1]. One important limiting factor is the high kernel moisture content (KMC) at harvest, which results in higher kernel breakage and an increased impurity rate. These limitations greatly increase the cost of harvest, reduce the economic benefit, and affect seed quality [2, 3]. According to previous research, 15%-25% KMC is the optimal level for mechanized harvest; however, in the main production area of China, the Huang-Huai-Hai region, the KMC ranged from 21.5%-33.1% with an average of 27.8% for summer maize [4]. Furthermore, decreased KMC in medium-maturing maize hybrids is considered to balance the KMC and yield for mechanized grain harvest, as the lower grain moisture mainly resulted from the > 30% dry matter in stover translocated to grain [5]. Therefore, adjusting the germplasm to produce low-KMC maize for improved mechanized harvest has become a research hotpot for breeders.

In recent decades, considerable scientific advances have been aided by understanding the regulatory mechanisms of kernel development related to kernel size, metabolism, and nutrient content including protein, starch, oil, and flavonoids [6–8]. Physiological analysis of kernel development has revealed that the process of kernel dehydration is divided into two stages: developmental water loss and physical dehydration [9]. The first stage is a grain filling stage, which is considered as physiological maturation due to the accumulation of dry matter. The second stage is the post-physiological maturity stage, during which the kernel undergoes rapid dehydration and gradually transitions into dormancy [10]. It was found that KMC and kernel dehydration rate (KDR) are related to growth period, and maize with shorter growth periods always have a faster dehydration rate [11]. Additionally, KMC and KDR have been shown to be affected by extraneous factors, including bract length, bract layer number, cob size, grain number per ear, and climate [2, 12].

In recent years, research investigating the genetic mechanisms influencing KMC and KDR has increased. KMC and KDR can be affected by the characterization of germplasm, cultivation method, and environment [13]. Nonetheless, it shows high broad-sense heritability (0.66–0.85), and multiple quantitative trait loci (QTL) that control KMC were identified using genome-wide linkage and association analysis [14–19]. For instance, 31 KMC QTL and 17 KDR QTL were identified in three environments using recombinant inbred lines constructed from the inbred lines 844 and 807 [20]. Meanwhile, seven single-nucleotide polymorphisms (SNPs) associated with KDR have been identified using a genome-wide association study (GWAS) of 309 inbred maize lines. Of these, one candidate gene (*Zm00001d047468*, *Zmapt1*) has been found to be expressed differently in maize inbred lines with different KDR [21]. Recently, 71 QTL that influence KMC were identified through a GWAS using 513 diverse inbred maize lines. *GRMZM5G805627* (*ZmGAR2*) and *GRMZM2G137211* (*ZmCRY1-9*) were confirmed as candidate genes for controlling KMC through a combination of genetic population analysis, transcription profiling, and gene editing [22]. Owing to the complex characterization of KMC and KDR, extensive research is required to understand the whole genetic basis.

Multiple omics analyses, including genomics, transcriptions, proteomics, and metabolomics, have been used to explore the regulatory mechanisms of kernel dehydration in crops. For instance, 11 proteins related to dehydration tolerance have been detected at seed maturity in maize using two-dimensional electrophoresis and mass spectrometry [23]. Yu et al. identified four oleosins and 76 stress/defense proteins in maize during the maturation stage (40–50 days after pollination, DAP), which may protect seeds from damage [24]. Chen et al. elucidated that the expression of late embryogenesis abundant (LEA) protein, heat shock protein (HSP), and serpins was increased during the dehydration stage using iTRAQ-based proteomics [25]. Although previous research has demonstrated significant progress regarding dehydration mechanisms, further work is required to clarify the regulatory mechanisms at the proteome and metabolome levels.

According to a recent study using time-resolved multiomics analysis to reveal the genetics of KMC and KDR in maize, 42 DAP was considered as a key time point for KMC transformation in late kernel development [26]. Data from production practice suggested that 35–49 DAP is when the dynamic processes from physiological maturity to dehydration occur [27]. Although association and linkage analyses are used to identify QTL and genes related to kernel dehydration, they only focus on the kernel moisture at harvest. Moreover, there are more ways to understand the related regulation mechanism, especially the dehydration rate. In the present study, we detected the proteome differences in three maize inbred lines (KB182(A), KB207(K), and KB020(B)) with differing KMC and KDR at 35, 42, and 49 DAP. Our objective was to identify protein datasets influencing KMC and KDR during the key dehydration period. Additionally, by integrating metabolome analysis, the aims were to 1) establish the relationship between metabolism and KMC and KDR, and 2) construct a preliminary regulation network for KMC and KDR to provide a resource for further understanding the molecular mechanisms to reduce maize KMC at harvest.

## Results

### Variation of kernel moisture content and kernel dehydration rate

**Fig. 1:**
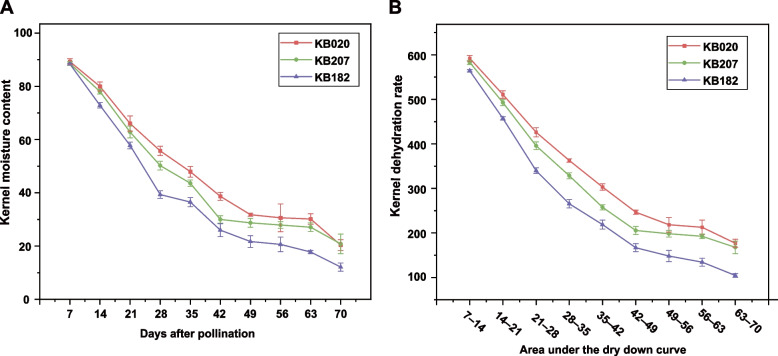
Variant of moisture content of different inbred lines in different periods. **A** Changes in moisture content of three inbred lines at 7–70 days after pollination. **B** Changes in AUDDC of three inbred lines at AUDDC1-AUDDC9

To reduce the effect of the environment on KMC and KDR, the sowing time was adjusted to ensure that the three inbred lines were pollinated on the same day. Then, the ears of the three maize inbred lines pollinated on the same day were used for KMC determination. All three inbred lines showed a trend for fast dehydration from 7 to 49 DAP, shifted to relative stability through 49 to 63 DAP, and then had a small fast decline in hydration from 63 to 70 DAP (Fig. 1A and Table S1). At the earlier stages, KMC was lowest in KB182 and highest in KB020. The variation trends of KB182 and KB020 were similar from 35 to 49 DAP, but both differed compared to the trend of KB207. The KMC of KB207 dropped sharply from 35 to 42 DAP; thereafter, it did not change markedly and ranged between 42 and 49 DAP. In addition, we evaluated KDR using area under the dry down curve (AUDDC) value as previously described [28]. KB182 had the fastest KDR, whereas KB020 had the slowest KDR among the three inbred lines in the whole-kernel development process. For KB207, the KDR is fast at 35 to 42 DAP and slow at 42 to 49 DAP, which resulted in its KMC being closer to that of KB020 at 35 and 49 DAP but differed significantly at 42 DAP when it was more similar to that of KB182 (Fig. 1B and Table S1). Therefore, the three inbred lines with different phenotype were suitable to study the mechanisms behind KMC and KDR.

### Proteome dynamics during the dehydration process

**Fig. 2:**
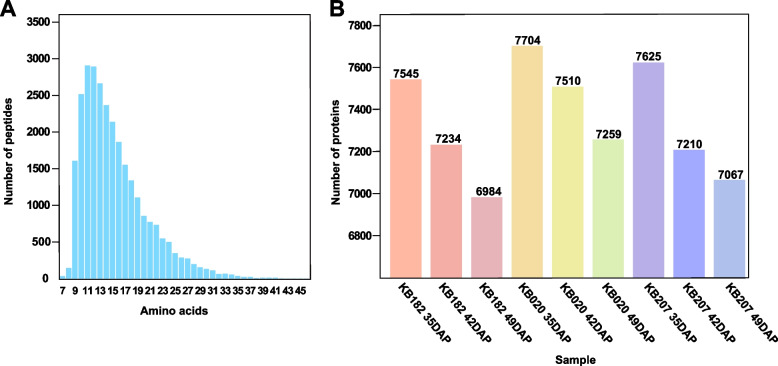
Basic information of all peptides and the number of identified proteins in all samples. **A** Distribution of peptide length in samples. **B** Distribution of proteins in samples

To explore the critical proteins in the key dehydration processes of maize seeds, we detected and quantified proteins in the kernels from KB182, KB020, and KB207 at 35, 42, and 49 DAP. Proteins were quantified based on data-independent acquisition (DIA) proteomics. In total, 28,372 peptides were detected, and most identified peptides were 9–19 amino acids in length (Fig. 2A). Of the 9,241 proteins identified, 8,358 of them were qualified, and among them, 96 proteins were sourced from Swiss-Prot and 8,262 from TrEMBL (Table S2). The number of identified proteins in each sample ranged from 6,984 to 7,704, and the number of identified proteins decreased along with the dehydration process (Fig. 2B). According to the principal component analysis (PCA), the three biological replicates of each sample were strongly correlated (Fig. S1). The protein abundances were validated based on parallel reaction monitoring (PRM) by selecting seven random proteins: catalase isozyme 3 (P18123), late embryogenesis abundant protein Lea14-A (B6UH99), granule-bound starch synthase 1b (A0A1D6HXP5), pathogenesis-related protein 10 (A0A1D6JZU3), HSP 26 (Q41815), L-ascorbate peroxidase (B4FWL1), and peroxidase (C0PKS1). The results of PRM were consistent with DIA data (Fig. S2). These results show that protein abundance qualification by DIA was highly effective and feasible.

### Identification and annotation of DEPs in different stage or different samples

**Fig. 3:**
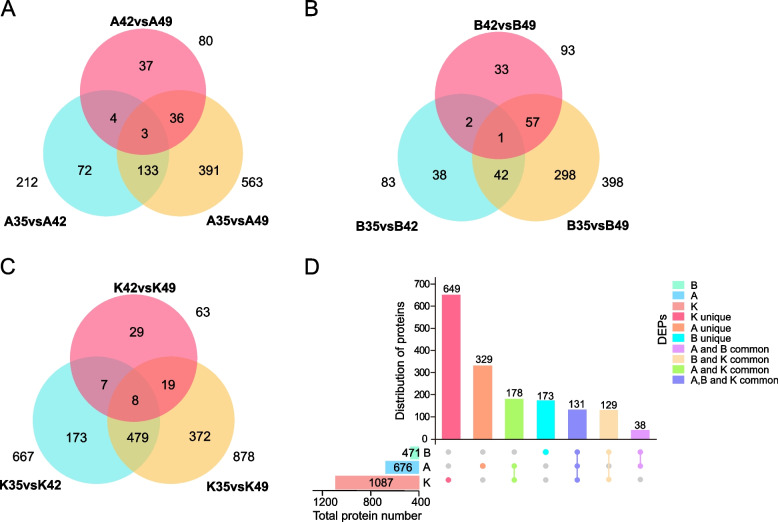
Comparison of differentially expressed proteins (DEPs) in each inbred line at different periods. **A** Number of DEPs of KB182 in three periods. **B** Number of DEPs of KB020 in three periods. **C** Number of DEPs of KB207 in three periods. **D** Distribution of DEPs in three inbred lines

To explore the important proteins for dehydration during different growth periods and in different inbred lines, we compared different inbred lines at the same time and the same inbred line at different periods using the standard with log_2_(fold change) > 1 or <  − 1 in expression and *P* value < 0.05. Finally, we obtained 2,779 different expressed proteins (DEPs) after deleting the repetitive DEPs (including all those shown in Tables S3 and S6). First, we compared the samples from the same inbred lines at different stages, which included nine comparison pairs: A35 *vs* A42, A42 *vs* A49, A35 *vs* A49, B35 *vs* B42, B42 *vs* B49, B35 *vs* B49, K35 *vs* K42, K42 *vs* K49, and K35 *vs* K49, where we defined KB182 as A, KB182 as B, and KB207 as K (Fig. 3A-C and Table S3). Then, 131 common DEPs were detected between the three inbred lines (Fig. 3D), which may play an identical role in regulating dehydration, and they were annotated in response to stress (including that from water, external stimulus, chemicals, desiccation, and oxidative stress) and growth (Table S4). Furthermore, 12 common proteins were detected in all comparative pairs whose functions include response to stress, carbohydrate metabolism, gene expression regulation, and material transport (Table S5).

**Fig. 4:**
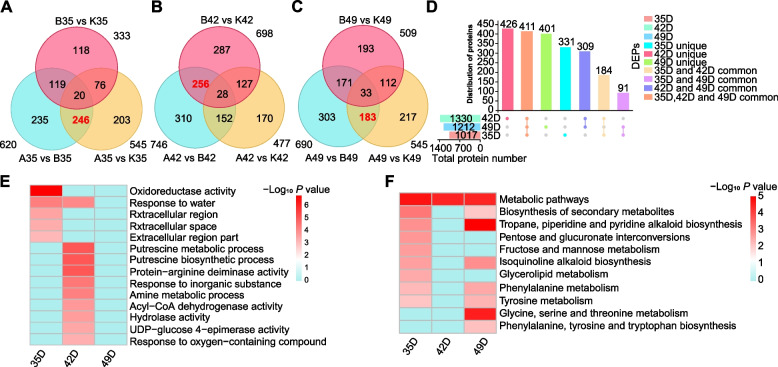
Comparison of differentially expressed proteins (DEPs) in different inbred lines in the same period. **A** Number of DEPs of three inbred lines at 35 days after pollination (DAP). **B** Number of DEPs of three inbred lines at 42 DAP. **C** Number of DEPs of three inbred lines at 49 DAP. **D** Distribution of DEPs in the three periods. **E** Gene Ontology enrichment results of key proteins after comparison. **F** Kyoto Encyclopedia of Genes and Genomes enrichment results of key proteins after comparison

Second, another nine pairs were obtained by comparing the samples from different inbred lines at the same stage of development: A35 *vs* B35, A35 *vs* K35, B35 *vs* K35, A42 *vs* B42, A42 *vs* K42, B42 *vs* K42, A49 *vs* B49, A49 *vs* K49, and B49 *vs* K49 (Fig. 4A-D and Table S6). The number of DEPs identified between KB182 and KB020 at all three stages was consistently higher than that identified between KB182 *vs* KB207 and KB207 *vs* KB020, which is consistent with the trends of KMC and KDR (Fig. 1). Therefore, we focused on KB182 and KB020 to further explore the key proteins and pathways related to dehydration. Comparative analysis showed that there were 620, 746, and 690 DEPs between KB182 and KB020 at 35 DAP, 42 DAP, and 49 DAP, respectively. There are 235 common DEPs, which may be closely related to dehydration at all time points (Table S7). At the same time, we performed Gene Ontology (GO) and Kyoto Encyclopedia of Genes and Genomes (KEGG) analyses on the DEPs at the three time points, and they were enriched at all time points, including response to inorganic substance, response to oxygen-containing compound, catalytic activity, metabolic pathways, biosynthesis of secondary metabolites, and carbohydrate metabolism (fructose and mannose metabolism, amino sugar and nucleotide sugar metabolism, and glycolysis/gluconeogenesis) (Table S8).

According to our previous division of dehydration for three inbred lines, to better understand the regulation of KMC, 246 common DEPs between the comparisons A35 *vs* B35 and A35 *vs* K35 were used for deeper analysis due to the KMC of KB020 and KB207 being closer but differing significantly from the KMC of KB182. Similarly, 256 common DEPs between the comparisons A42 *vs* B42 and B42 *vs* K42 and 183 common DEPs between the comparisons A49 *vs* B49 and A49 *vs* K49 are also considered key proteins. These DEPs were mainly enriched in response to water, oxidoreductase activity, fructose and mannose metabolism, pentose and glucuronate interconversions, biosynthesis of secondary metabolites, and glycine, serine, and threonine metabolism (Fig. 4E, F and Table S9).

### Relationship of protein abundance with KMC and KDR

**Fig. 5:**
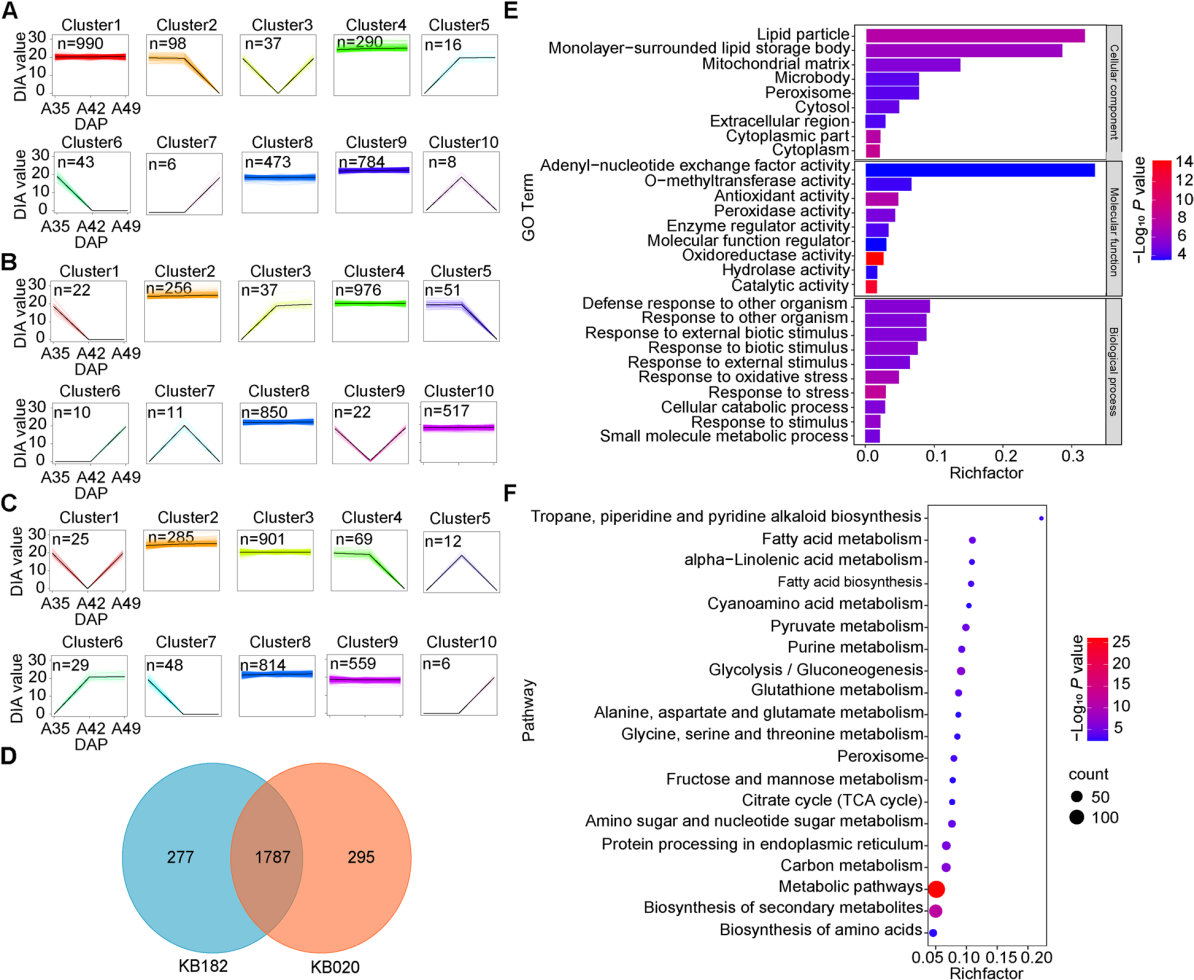
Expression patterns of three inbred lines at three time points. **A** Protein expression pattern of KB182. **B** Protein expression pattern of KB020. **C** Protein expression pattern of KB207. **D** Venn diagram of high-expression proteins in KB182 and KB020. **E** Gene Ontology enrichment results of 1,787 high-expression proteins. **F** Kyoto Encyclopedia of Genes and Genomes enrichment results of 1,787 high-expression proteins

To determine the relationship between protein abundance and KMC phenotype, we compared the curves of observed dehydration and protein abundance using K-means clustering with the quantified protein (Fig. 5A-C). Proteins were then divided into 10 clusters for each of the three inbred lines. For KB182 and KB020, the parallel dehydration curves showed fast dehydration from 35 to 49 DAP; therefore, we focused on the clusters 1, 4, and 9 of KB182 and clusters 2, 4, and 8 of KB020 because they showed a high expression abundance at these three time points (Fig. 5A, B). In total, 1,787 DEPs were found between KB182 and KB020 that were enriched in response to stress (including oxidative stress, and response to stimulus and water), small molecule metabolic processes, antioxidant activity, fatty acid metabolism, carbohydrate metabolism (glycolysis/gluconeogenesis, fructose and mannose metabolism, pentose phosphate pathway, and starch and sucrose metabolism), carbon metabolism, and glutathione metabolism (Fig. 5D-F and Table S10). For KB207, whose dehydration was fast from 35 to 42 DAP but slow from 42 to 49 DAP, a similar trend was observed in cluster 4 with 69 DEPs, which were associated with oxidation reduction (*P* = 1.10E-08 and FDR = 4.90E-07) in the GO analysis.

**Fig. 6:**
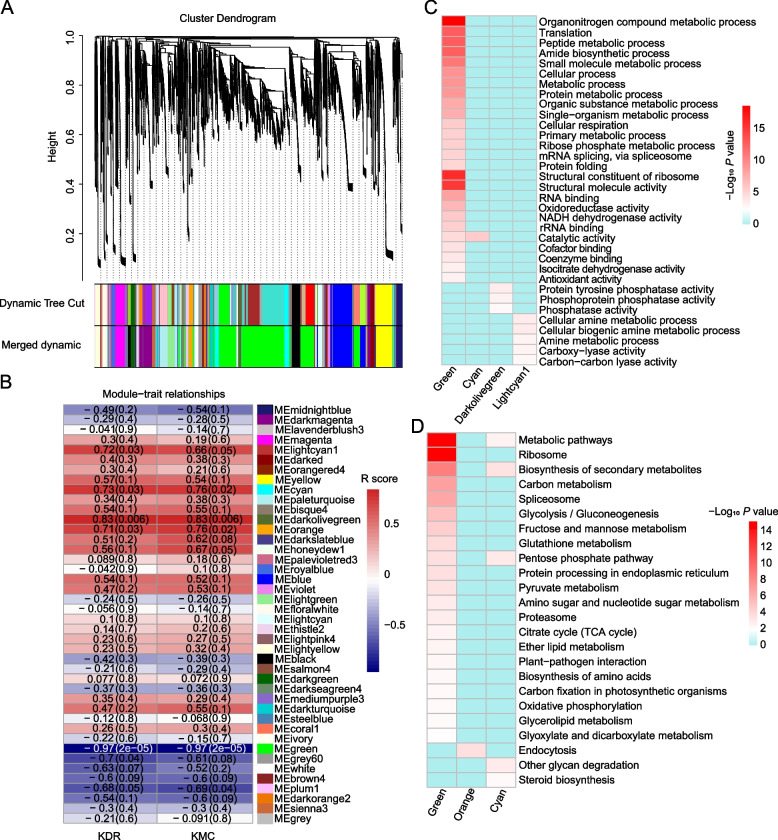
Weighted gene co-expression network analysis identified a dehydration-related module. **A** Protein expression module. **B** Relationships between the modules and dehydration. **C** Gene Ontology enrichment results of important modules. **D** Kyoto Encyclopedia of Genes and Genomes enrichment results of important modules

**Fig. 7:**
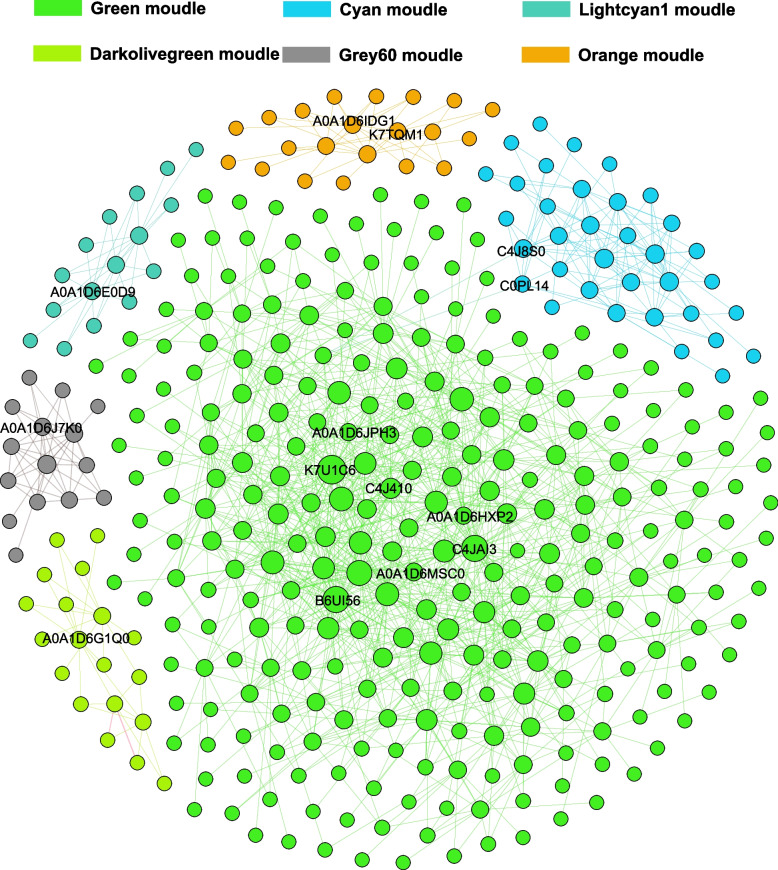
Protein–protein interaction network of hub proteins (Top 5%)

We systematically investigated the relationship between protein abundance and dynamic KMC using the correlation weighted values between different protein expressions with weighted gene co-expression network analysis (WGCNA) (Fig. 6A). All expressed proteins were divided into 42 modules (Table S2), in which six modules were significantly correlated with KMC and KDR using the thresholds of R > 0.6 and *P* < 0.05, including light cyan (65), cyan (259), orange (115), dark olive green (88), green (2469), and gray (133) (Fig. 6B). These proteins were found to participate in response to abiotic stimuli, small molecule metabolic processes, amide biosynthesis, carbohydrate metabolism (the pentose phosphate pathway, glycolysis/gluconeogenesis, citrate cycle, and fructose and mannose metabolism), glutathione metabolism, and biosynthesis of secondary metabolites, among others (Fig. 6C, D and Table S11). An interaction network was constructed using proteins of the top 5% connectivity as hub proteins in important modules (Fig. 7 and Table S12). This network showed that the unique interaction between the cyan and green modules was linked by C4J8S0 and C0PL14. These hub proteins were involved in antioxidant systems and carbohydrate, energy, and lipid metabolism. In addition, these hub proteins were mainly enriched in response to water, organonitrogen compound metabolic processes, cellular amide metabolic processes, and ribosomes (Table S13).

### Variation in metabolic components in KB182 and KB020

**Fig. 8:**
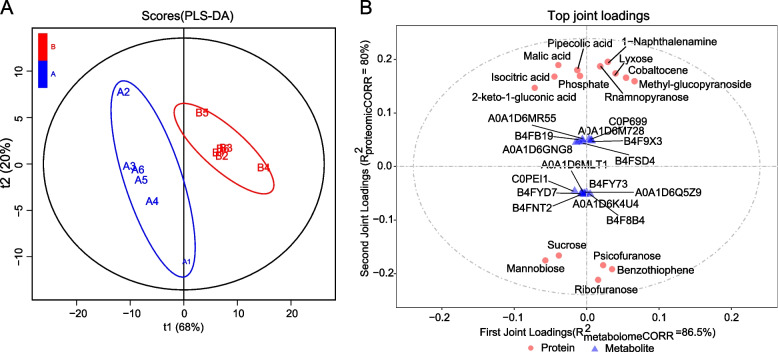
Metabolome analysis between KB182 and KB020. **A** Partial least-square discriminant analysis (PLS-DA) score plot of samples in different lines. **B** Top 15 most closely associated metabolites and proteins

Based on phenotypic results, we found a significant difference in KMC between KB182 and KB020 when it reached its maximum at 42DAP during our proteomic testing of the three stages (Fig. 1). Moreover, the number of DEPs in A42 *vs* B42 was the highest among different inbred lines compared in the three stages. Therefore, we chose KB182 and KB020 with 42DAP for metabolomic analysis.A total of 151 metabolites were detected, and the metabolic components in these two inbred lines showed considerable sample separation (Fig. 8A). Of these, 18 metabolites had significantly different abundance and were mainly associated with carbohydrate, amino acid, and energy metabolism (Table S14).

By integrating the metabolome and proteome analyses using two-way orthogonal partial least square with discriminant analysis (O2PLS), the joint parts (R2 proteomicCORR and R2 metabolomeCORR) indicated that more than 80% of the metabolomic and proteomic variation was explained. The most closely associated metabolites and proteins included ubiquitin carrier protein, aldolase-type TIM barrel family protein, nonspecific lipid-transfer protein, and germin-like protein. Metabolites with strong correlation with proteins included sucrose, isocitric acid, phosphate, and malic acid (Fig. 8B).

## Discussion

With the increasing population and decreasing labor, agricultural production has become more intensive and mechanized, thus requiring maize varieties suitable for kernel-mechanized harvesting, which can save both time and labor. KMC and KDR are important factors affecting mechanized maize harvesting [29, 30]. According to precious study, KB182, KB207 and KB020 showed significant differences in KMC and KDR, which were bred from the Shaan B group. The KDR of KB182 is fast throughout the whole dehydration process, and the KMC is always low. In contrast the KDR of KB020 is slow throughout the dehydration process, and the KMC is always high. However, KB207 has a rapid decrease in KDR at 35–42 DAP and then tends to stop. The differences between KMC and KDR make them very suitable for studying kernel dehydration. Additionally, the previous study only analyzes KMC at the harvest stage and focuses on gene variation [15, 16]. Proteins and metabolites have not been explored yet, and the related metabolic pathways are still unclear. Therefore, we coordinated pollination on the same day to minimize environmental noise and compared the differences in protein and metabolism abundance to explore key proteins and metabolites and understand the regulatory mechanisms of KMC and KDR.

In this study, 2,779 DEPs were identified that may be involved in regulating kernel dehydration, and 12 of them were identified at all stages of different inbred lines, which may play a role throughout all stages of dehydration. At the same time, we identified the metabolomics of KB182 and KB020 at 42DAP to understand the relevant mechanisms affecting kernel dehydration from the perspective of metabolites. We detected a total of 18 differential metabolites, of which 7 were carbohydrates, and 6 were amino acids, indicating that carbohydrates and amino acids may be the main reasons for the differences in KMC and KDR between KB182 and KB020.Futhermore, we compared DEPs with the QTL and SNP mapped by previous research [31–36]. Finally, 441 DEPs were identified as candidate proteins. (Table S15). These results provide the possibility for subsequent understanding regulation mechanism of kernel dehydration.

As products of plant life activities, reactive oxygen species (ROS) can participate in signal transduction, growth, and development but are accumulated excessively in seeds during the rapid dehydration stage, which causes irreversible cell damage [37]. Therefore, the effective operation of antioxidant systems is crucial for the seed to resist damage during dehydration [38, 39]. We observed the enrichment of eight main antioxidant system enzymes, including five peroxidases (B4F7T9, C0PKS1, B4FBH0, C0HEE6, and C0P813), two catalases (P18123 and P12365), and one superoxide dismutase (B1PEY4), in our inbred maize lines. The same phenomenon was observed in a recent study in which peroxidase and superoxide dismutase were phosphorylated, and their activity was decreased after severe dehydration stress; however, this did not seem to be sufficient to eliminate ROS and lead to increased H_2_O_2_ levels [40]. The ascorbate–glutathione cycle is another important regulator of H_2_O_2_ scavenging. We found three key proteins located in the green and cyan modules, including dehydroascorbate reductase (C0P9V2), which degrades docosahexaenoic acid to ascorbic acid, and glutathione reductase (B4FWU6 and A0A1D6JPH3), a catalyst for the regeneration of glutathione [41]. These proteins might affect kernel dehydration via ascorbate–glutathione cycle regulation, which has not been reported to date.

Previous studies considered that carbohydrates participate in the protection of seeds in two ways: water substitution and vitrification of water phase [42, 43]. Trehalose was first found to play an important role in plant dehydration, but subsequent studies found that sucrose plays a similar role [44]. Raffinose-family oligosaccharides accumulate during seed maturation and play important roles in seed vigor [45]. In addition, the raffinose:sucrose ratio can influence membrane stability and protect high-moisture seeds from dehydration in maize [46]. The present study indicated that sucrose synthase (A0A1D6P836, C0P6F8) and sucrose phosphate synthase (A0A1D6N358) were more abundant in high-moisture inbred lines and during the early stages of growth. Previous studies have shown that the expression of glucose and fructose first increased, then decreased, and finally disappeared during seed development [47]. This indicates that glucose and fructose do not play the same role as sucrose in the process of dehydration, and they may be used as raw materials to synthesize sucrose [48]. Similarly, we observed an accumulation of fructose, glucose, and related proteins, and a series of enzymes related to starch metabolism (B4FYM6, Q5NKP6, A0A1D6K8T3, Q9SYS1, and B5AMJ8) in the inbred lines. These results indicated that carbohydrates, especially raffinose and sucrose, can regulate kernel dehydration (Table S16).

Previous studies have divided the protective proteins related to dehydration into two categories: LEA proteins and HSPs. These proteins are activated by abscisic acid (ABA), accumulate at the later stage of seed embryo development, are abundant in dry seeds, and play defensive and protective roles in seed dehydration [49–51]. In the present study, five LEA proteins, encoded by *Zm00001d009382, Zm00001d034002, Zm00001d038870, Zm00001d043709,* and *Zm00001d017021,* showed higher expression later in kernel development, which is consistent with the results of others [25, 52]. In addition, their abundance was highest in KB182 and lowest in KB020 of the same stage, indicating that a higher LEA protein abundance might cause faster dehydration. One HSP (*Zm00001d015777*) was identified as a DEP in this study, which was related to the kernel dehydration trend and LEA protein expression. These results indicate that LEA protein and HSPs play a crucial role in maize kernel dehydration and may regulate kernel dehydration through a similar mechanism in maize; however, further research is required to validate this.

**Fig. 9:**
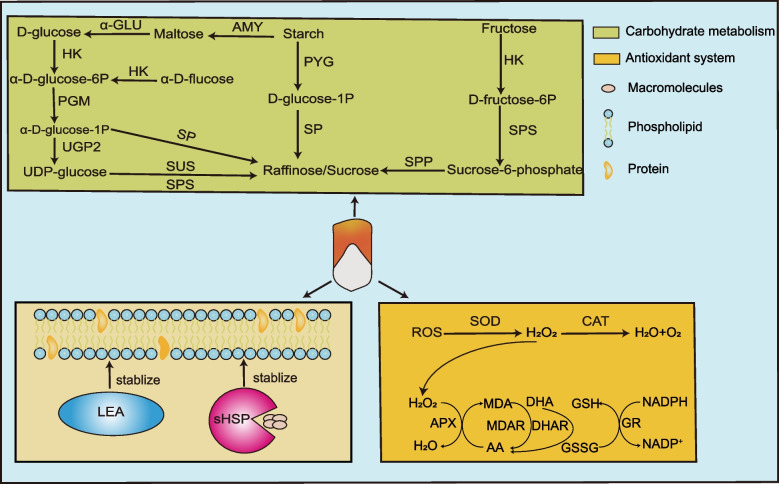
Role of different types of protein during maize kernel dehydration. AA (ascorbic acid); ROS (reactive oxygen species); APX (ascorbate peroxidase); CAT (catalase); DHA (dehydroascorbate); DHAR (dehydroascorbate reductase); GR (glutathione reductase); GSH (glutathione); GSSG (glutathione); H_2_O_2_ (hydrogen peroxide); MDA (monodehydroascorbate); MDAR (monodehydroascorbate reductase); NADPH (nicotinamide dinucleotide phosphate); SOD (superoxide dismutase); AMY (α-amylase); α-GLU (glucosidase); HK (hexokinase); SPS (sucrose-phosphate synthase); SP (sucrose phosphorylase); SPP (sucrose-6-phosphatase); PYG (glucan phosphorylase); PGM (phosphoglucomutase); UGP2 (UTP–glucose-1-phosphate uridylyltransferase); sHSP (small HSP); LEA (late embryogenesis abundant) protein

According to our integrative analysis of the proteome and metabolome, we propose a model of how LEA proteins, HSPs, antioxidant systems, and carbohydrate metabolism (raffinose and sucrose) are involved in regulating kernel dehydration (Fig. 9). In particular, when the kernel enters the stage of rapid dehydration, ABA synthesis is activated after the dehydration pressure is felt inside the kernel. ABA induces the expression of LEA proteins and HSPs in response to kernel dehydration [53]. At the same time, the dynamic balance of ROS production and scavenging is disrupted, and the antioxidant system begins to work effectively to remove ROS. However, in the rapid dehydration stage, this defense method is gradually weakened, and sucrose metabolism replaces water molecules to compensate for the deficiency of the antioxidant system and maintain cell membrane stability [54]. A limitation of our study was that we were unable to verify the function of the nominated protein at the molecular level. However, the results we obtained by minimizing the environmental noise are helpful to understand the molecular mechanism of kernel dehydration and provide a theoretical basis for mechanized-harvest breeding. In future, more detailed work is necessary to understand the genetic basis through traditional map-based cloning and validate some core genes through gene editing for breeding improved maize.

## Methods

### Field experiment and phenotyping

Inbred lines KB182, KB020, and KB207 were bred from the Shaan A and Shaan B groups by the maize biology and genetic breeding group at Northwest A&F University, Shaanxi, China. To ensure that all ears were pollinated on the same day and minimize environmental noise, the three inbred lines KB182, KB207, and KB020 were sown on May 11th, 19th, and 17th in 2019, respectively. All field experiments were conducted with three replications in the Yangling maize base of Northwest A&F University. After unified pollination on July 25th, 100 kernels were collected from each ear at 10 successive stages (7, 14, 21, 28, 35, 42, 49, 56, 63, and 70 DAP). After sampling, kernel fresh weight (W1) was measured with a 0.001 g digital scale. Then, the samples were heated in an oven at 105℃ for 30 min and finally dried at 70℃ to constant weight (W2). The formulae for KMC and area under the dry down curve used were as previously described [26, 28] and are as follows:

$${\text{KMC}}(\mathrm{\%}) = [({\text{W}}1 - {\text{W}}2)/{\text{W}}1] \times 100\mathrm{\%},$$

Where W1 is the kernel fresh weight, and W2 is the final weight after drying. According to KMC, KDR was calculated using the AUDDC method using the following formula:

$$\text{AUDDC } = {\sum }_{{\text{i}}}^{1}\left[\left({{\text{KMC}}}_{{\text{i}}}+{{\text{KMC}}}_{{\text{i}}+1}\right)/2\right]\left({{\text{t}}}_{{\text{i}}+1}-{{\text{t}}}_{{\text{i}}}\right),$$

Where KMC is the moisture content of the kernel,$i$ is the $ith$ measured time, and ${t}_{i}$ is the corresponding day after pollination (7, 14, 21, 28, 35, 42, 49, 56, or 70).

Nine AUDDC traits were established based on the phenotype at 10 time points: AUDDC1 (7–14 DAP), AUDDC2 (14–21 DAP), AUDDC3 (21–28 DAP), AUDDC4 (28–35 DAP), AUDDC5 (35–42 DAP), AUDDC6 (42–49 DAP), AUDDC7 (49–56 DAP), AUDDC8 (56–63 DAP), and AUDDC9 (63–70 DAP). The lower the AUDDC, the faster the KDR.

### Protein extraction and digestion

According to the variation in KMC, samples taken on 35, 42, and 49 DAP from KB182, KB020, and KB207 were used for protein extraction and analysis; these samples were named accordingly (A35, A42, A49, B35, B42, B49, K35, K42, and K49), where A is KB182, B is KB020 and K is KB207. Three biological replicates of the samples at each time point were mixed as a pooled sample. Protein extraction, peptide preparation, and quantification of each sample were conducted in triplicate using mass spectrum (MS) analysis by PTM Biolab LLC based on DIA technology as previously described [55]. The sample was first ground with liquid nitrogen, and then the powder was transferred to a 5-mL centrifuge tube and sonicated for 3 min on ice using a high-intensity ultrasonic processor (Scientz) in lysis buffer. An equal volume of Tris-saturated phenol (pH 8.0) was added. Then, the mixture was further vortexed for 5 min. After centrifugation (4 °C, 10 min, 5500 g), the upper phenol phase was transferred to a new centrifuge tube. Proteins were precipitated by adding at least four volumes of ammonium sulfate-saturated methanol and incubated at − 20 °C for at least 6 h. After centrifugation at 4 °C for 10 min, the supernatant was discarded. The remaining precipitate was washed thrice with ice-cold methanol, followed by ice-cold acetone. The protein was redissolved in 8 M urea.

The sample was slowly added to the final concentration of 20% (m/v) trifluoroacetic acid to precipitate protein, then vortexed to mix and incubated for 2 h at 4 °C. The precipitate was collected by centrifugation at 4500 g for 5 min at 4 °C. The precipitated protein was dissolved in 200 mM triethyl-ammonium bicarbonate buffer and ultrasonically dispersed. Trypsin was added at 1:50 trypsin-to-protein mass ratio for the first digestion overnight. The sample was reduced with 5 mM dithiothreitol for 60 min at 37 °C and alkylated with 11 mM iodoacetamide for 45 min at room temperature in darkness. Finally, the peptides were desalted by the Strata X SPE column.

### Spectral library building—LC–MS/MS analysis

The tryptic peptides were dissolved in solvent A (0.1% formic acid, 2% acetonitrile), and directly loaded onto a homemade reversed-phase analytical column. Peptides were separated with a gradient from 4 to 32% solvent B (0.1% formic acid in 90% acetonitrile) over 114 min, 32% to 80% in 3 min, and holding at 80% for the last 3 min. The separated peptides were analyzed in data-dependent acquisition (DDA) mode by Q ExactiveTM HF-X (Thermo Fisher Scientific) with a nano-electrospray ion source.

### Data-independent acquisition

The iRT kit was added to all the samples according to the manufacturer’s instructions. The LC gradient was kept consistent with those in the spectral library building method. The separated peptides were analyzed in Q ExactiveTM HF-X (Thermo Fisher Scientific) with a nano-electrospray ion source. The data acquisition was performed in DIA mode. Each cycle contains one full scan followed by 70 DIA MS/MS scans with a predefined precursor m/z range. The HCD fragmentation was performed at a normalized collision energy of 27%.

All DIA data were analyzed in Skyline (v 20.1.0). The DDA search results were imported to Skyline to generate the spectral library, and the retention times were aligned to iRT reference values. Relative quantification of proteins was performed using the MSstats package. PCA was performed to evaluate the repeatability of all samples using quantified proteins. To confirm DIA and data-dependent analysis data, 24 proteins were randomly selected and quantified using PRM analysis performed by PTM Biolab LLC. DEPs were identified according to the standard with *P* value < 0.05 and log_2_(fold change) > 1 or <  − 1.

### Non-targeted metabolic profiling using GC–MS

Based on the variation in KMC and proteomics analyses, KB182 and KB020 with different KMC and KDR values were used for metabolic analysis at 42 DAP. Metabolic analysis was determined using a gas chromatograph-mass spectrometer (GC–MS; 7890A-5975C, Agilent Technologies, Palo Alto, CA, USA), and each sample was analyzed with two biological and three technical replicates. The samples pre-cooled in liquid nitrogen were ground using a Mixer/mill (MM400; Retsch) with a steel ball for 30 s at 30 HZ. Fifty milligrams of *Platycerium wallichii* Hook. powder of each sample was extracted following the procedures described in a previous study [56–58]. The extract was centrifuged at 23,128 g for 10 min at 4 °C. The fixed volume of 200 μL of the polar phase was transferred into a pre-labeled 1.5-mL microcentrifuge tube. Then, the samples were dried in a SpeedVac concentrator without heating. The dried 200 μL aliquots from the lower phase for primary metabolite profiling were derivatized with N-methyl-N-(trimethylsilyl) trifluoroacetamide as described previously [59] and further analyzed using GC–MS (7890A-5975C, Agilent, USA). One µL was taken from each sample and injected into GC–MS at 270 °C in a split mode (50:1) with helium carrier gas (> 99.999% purity) flow set to 1 mL/min and separated by a DB-35MS UI (30 m × 0.25 mm, 0.25 µm) capillary column. The temperature was isothermal for 4 min at 90 °C, followed by an 8 °C increase per minute ramp up to 205 °C, then held constant for 2 min, and finally ramped up at a rate of 15 °C per minute to 310 °C and held constant for 2 min. The transfer line temperature was set to 300 °C, and the ion source temperature was set to 230 °C. The mass range analyzed was from m/z 85 to 700. The Agilent MassHunter Qualitative Analysis software version B.06.00 (Agilent Technologies, Palo Alto, CA, USA) and Agilent MassHunter Quantitative Analysis software version B.07.01 (Agilent Technologies, Palo Alto, CA, USA) were both used for GC–MS data analyses. The NIST library and in-house database established using authentic standards were used together for metabolite identification. Supervised partial least-square (PLS) discriminant analysis was performed to construct a high level of group separation. To obtain an overview of the model, data were fit to highlight discriminant metabolites. The variable importance in projection > 1 and *P* value < 0.05 were selected to determine significantly different metabolites between different comparison groups.

### Bioinformatics analysis

In order to further describe the DEPs, the TBtools (v1.0692) software was used to generate Venn diagrams [60]. The qualified proteins were clustered using the K-means cluster function in R (http://www.r-project.org/). The WGCNA was performed using the “WGCNA” package (v3.6.2) in R to determine the core expression protein modules [61]. A co-expression network was constructed via Gephi (v0.9.2) using the identified proteins related to KMC and KDR with the thresholds of r > 0.6 and *P* < 0.05 [62]. MetaboAnalyst (http://www.metaboanalyst.ca) was used to enrich important metabolite pathways [63]. The O2PLS model (https://www.omicshare.com/tools) was used to determine related metabolites and proteins by integrating proteomic and metabolome data. Finally, the core proteins were enriched through GO analysis using the agriGO web server (http://bioinfo.cau.edu.cn/agriGO/index.php) and KEGG pathway enrichment analysis with the Kobas web server (http://kobas.cbi.pku.edu.cn/) [64, 65]. FDR < 0.05 was used as the threshold to obtain significantly enriched GO terms and pathways.

### Supplementary Information

**Additional file 1: Figure S1.**Two-dimensional scatter plot of principal component analysis (PCA) distribution of all samples using quantified proteins.

**Additional file 2: Figure S2.**Parallel reaction monitoring validation of several proteins identified by the DIA data.

**Additional file 3: Table S1.  **KMC and AUDDC phenotype data of three inbred lines.

**Additional file 4: Table S2. **DIA quantitative information.

**Additional file 5: Table S3. ** Information of differentially expressed proteins in different periods of the same inbred line.

**Additional file 6: Table S4. **The common proteins detected in three inbred lines.

**Additional file 7: Table S5. **The common proteins detected in different periods at same inbred line.

**Additional file 8: Table S6. **Information of differentially expressed proteins of different inbred lines in the same period.

**Additional file 9: Table S7. **The common proteins detected in KB182 VS KB020 at three periods.

**Additional file 10: Table S8. **GO and KEGG enrichment of key proteins in all comparison group.

**Additional file 11: Table S9. **GO and KEGG enrichment of KB182 VS KB020 at three periods.

**Additional file 12: Table S10. **GO and KEGG enrichment of proteins with the same expression pattern of KB182 and KB020.

**Additional file 13: Table S11. **GO and KEGG enrichment in different modules.

**Additional file 14: Table S12. **TOP 5% of hub protein identified by WGCNA.

**Additional file 15: Table S13. **GO and KEGG enrichment of hub protein identified by WGCNA.

**Additional file 16: Table S14. **GC-MS metabolite identification results.

**Additional file 17: Table S15. **Proteins significantly associated with KMC and KDR turough compare with previous study.

**Additional file 18: Table S16. **Sugars significantly associated with dehydration.

## Data Availability

The data reported in this paper have been deposited in the OMIX, China National Center for Bioinformation/Beijing Institute of Genomics, Chinese Academy of Sciences (https://ngdc.cncb.ac.cn/omix: accession no.OMIX001238).

## Abbreviations

DAP: Days after pollination
KDR: Kernel dehydration rate
KMC: Kernel moisture content
QTL: Quantitative trait loci
SNPs: Single-nucleotide polymorphisms
GWAS: Genome wide association study
iTRAQ: Isobaric tags for relative and absolute quantification
A: KB182
K: KB207
B: KB020
AUDDC: Area under the dry down curve
PCA: Principal component analysis
PRM: Parallel reaction monitoring
GO: Gene ontology
KEGG: Kyoto encyclopedia of genes and genomes
WGCNA: Weighted gene co-expression network analysis
O2PLS: Two-way orthogonal partial least square with discriminant analysis
PLS-DA: Partial least-square discriminant analysis
ROS: Reactive oxygen species
LEA: Late embryogenesis abundant
HSP: Heat shock protein
ABA: Abscisic acid
AA: Ascorbic acid
APX: Ascorbate peroxidase
CAT: Catalase
DHA: Dehydroascorbate
DHAR: Dehydroascorbate reductase
GR: Glutathione reductase
GSH: Glutathione
GSSG: Glutathione
H_2_O_2_: Hydrogen peroxide
MDA: Monodehydroascorbate
MDAR: Monodehydroascorbate reductase
NADPH: Nicotinamide dinucleotide phosphate
SOD: Superoxide dismutase
AMY: α-Amylase
α-GLU: Glucosidase
HK: Hexokinase
SPS: Sucrose-phosphate synthase
SP: Sucrose phosphorylase
SPP: Sucrose-6-phosphatase
PYG: Glucan phosphorylase
PGM: Phosphoglucomutase
UGP2: UTP–glucose-1-phosphate uridylyltransferase
sHSP: Small heat shock protein
MS: Mass spectrum
DIA: Data-independent acquisition
GC–MS: Gas chromatograph mass spectrometer
