# Supplementary material for: Reveal the kernel dehydration mechanisms in maize based on proteomic and metabolomic analysis
Source: BMC Plant Biol. 2024 Jan 2;24:15. doi: 10.1186/s12870-023-04692-z (PMC10759482; doi:10.1186/s12870-023-04692-z)
Supplement: Supplementary file 1 — Additional file 1: Figure S1.Two-dimensional scatter plot of principal component analysis (PCA) distribution of all samples using quantified proteins. [file 12870_2023_4692_MOESM1_ESM.docx]

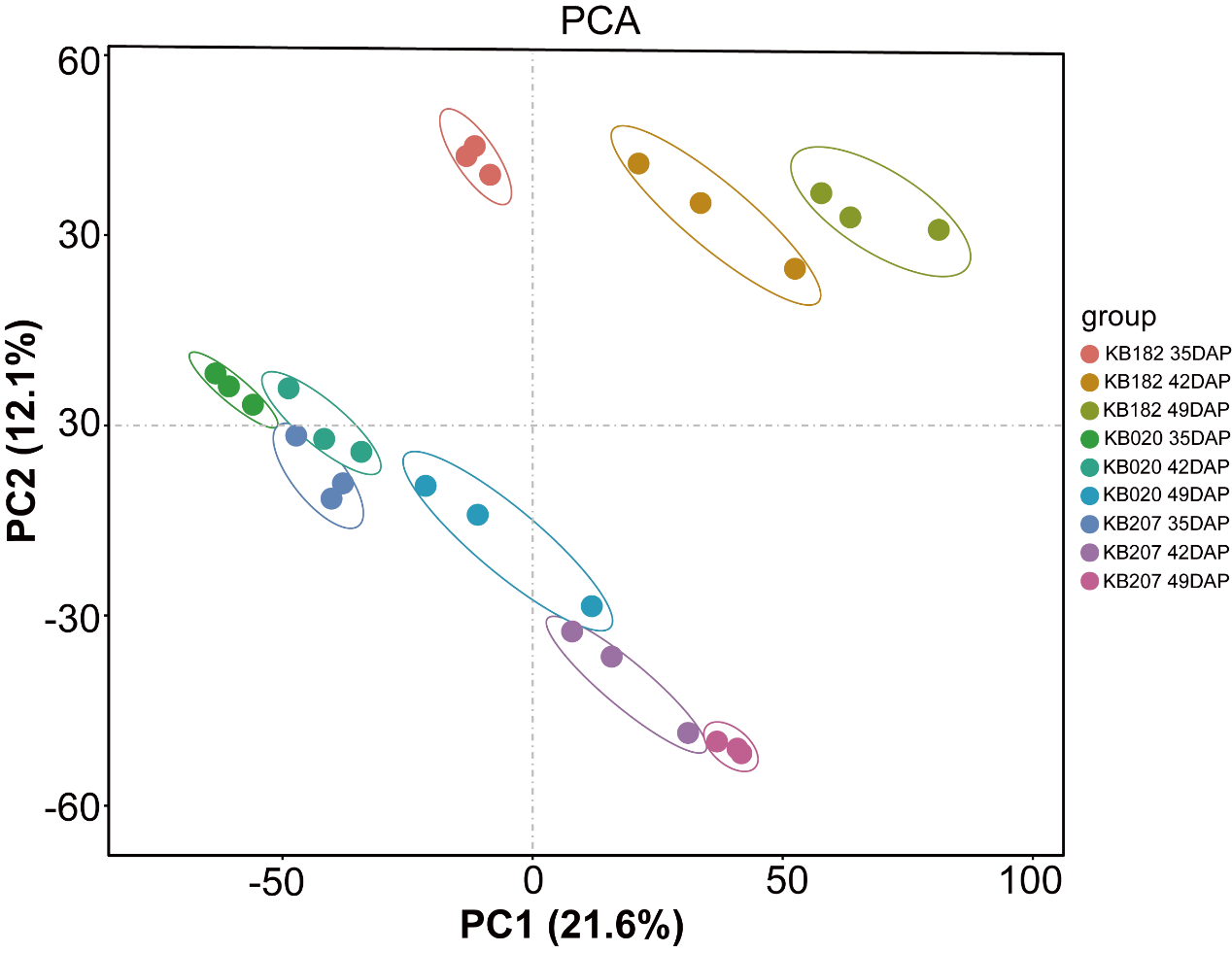


**Figure S1.** Two-dimensional scatter plot of principal component analysis (PCA) distribution of all samples using quantified proteins.
