# Supplementary material for: Reveal the kernel dehydration mechanisms in maize based on proteomic and metabolomic analysis
Source: BMC Plant Biol. 2024 Jan 2;24:15. doi: 10.1186/s12870-023-04692-z (PMC10759482; doi:10.1186/s12870-023-04692-z)
Supplement: Supplementary file 2 — Additional file 2: Figure S2.Parallel reaction monitoring validation of several proteins identified by the DIA data. [file 12870_2023_4692_MOESM2_ESM.docx]

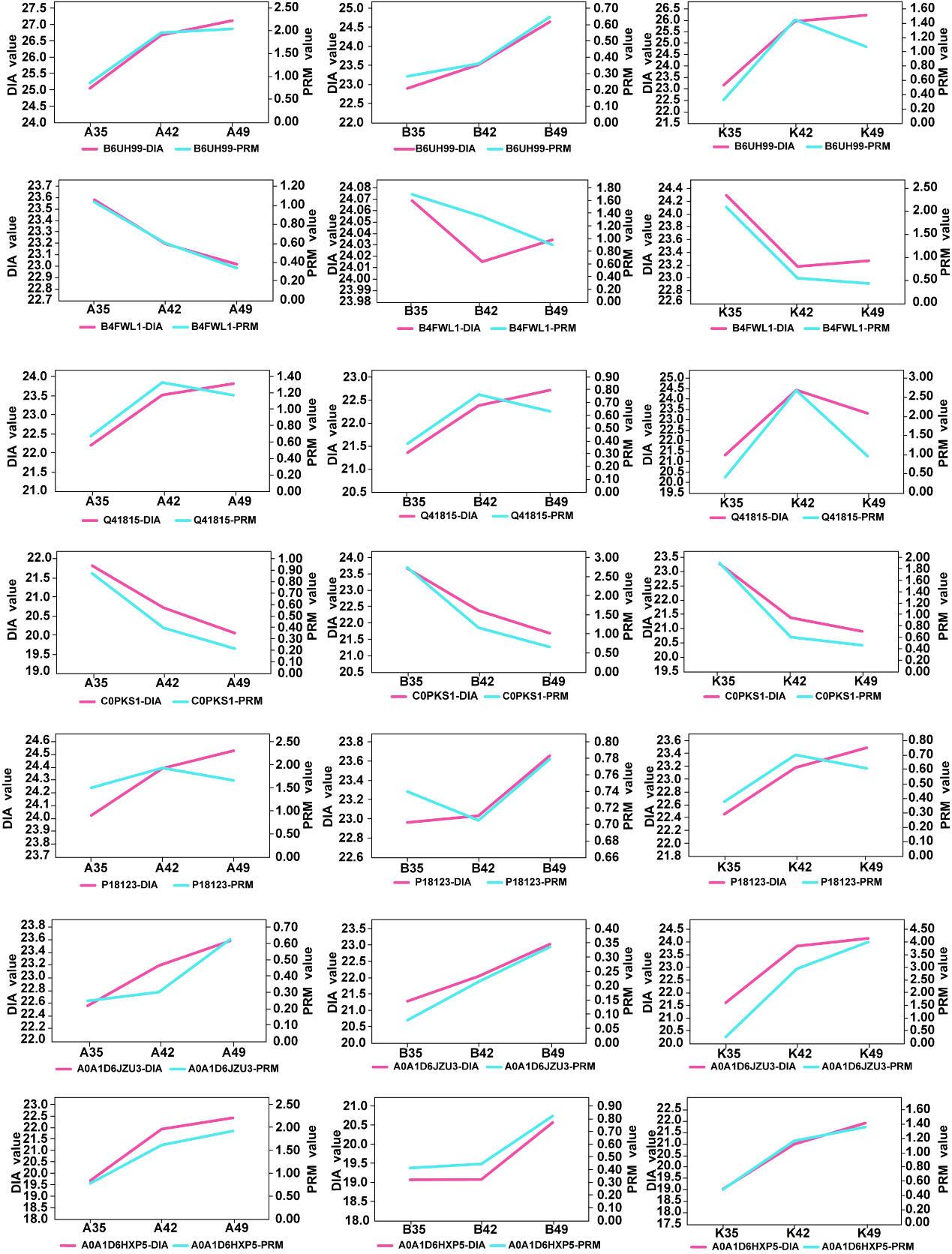


**Figure S2.** Parallel reaction monitoring validation of several proteins identified by the DIA data
